# Applicability of a green nanocomposite consists of reduced graphene oxide and β-cyclodextrin for electrochemical tracing of methadone in human biofluids validated by international greenness indexes

**DOI:** 10.1016/j.heliyon.2024.e40505

**Published:** 2024-11-19

**Authors:** Sayyed Esmaeil Moradi, Ardeshir Shokrollahi, Faezeh Shahdost-Fard

**Affiliations:** a Chemistry Department, Yasouj University, Yasouj, 75918-74831, Iran; b Department of Chemistry Education, Farhangian University, P.O. Box 14665-889, Tehran, Iran

**Keywords:** Methadone, Reduced graphene oxide, β-cyclodextrin, Greenness index, Non-invasive analysis, Human biofluids

## Abstract

**Background:**

Detecting methadone (MET) is crucial due to its severe side effects.

**Method:**

Herein, a green nanocomposite based on reduced graphene (rGO) and β-cyclodextrin (β-CD) has been introduced to modify a glassy carbon electrode (GCE) for real-time measurement of MET. This eco-friendly sensing interface has synergistically benefited from both advantages of rGO and β-CD including excellent electron transfer tunneling and surface area enhancement to selectively trap MET based on its shape and size.

**Significant findings:**

The developed sensor electrochemically detected MET at 0.8 V in buffer phosphate with a pH value of 7 under a wide linear concentration range (1 μM–830 μM), including a MET concentration level alarmed based on the consumer opioid tolerance according to the WHO's report. The limit of detection and analytical sensitivity values were calculated to be 333.33 nM and 0.0502 μA μM^−1^. The acceptable performance of the sensor to detect MET in various real human biofluids including serum, urine, and saliva samples, which is a bonus for the real-time and on-site measurement of MET, may open up a route for noninvasive routine tests in clinical samples. Moreover, the greenness profile of this strategy has been well evaluated by two common international metrics.

## Highlights

• The green nanocomposite containing rGO@β-CD **was used as the sensing interface.**
• The sensor detected MET from 7 μM to 830 μM under green chemistry principles.
• Sensor real-timely detected MET in serum, urine and saliva with good efficiency.
• The greenness of the applied method has been well evaluated by the international indexes.
• Strategy may open up a route for fast and noninvasive test of MET for real samples.

## Graphical abstract

**Figure:**
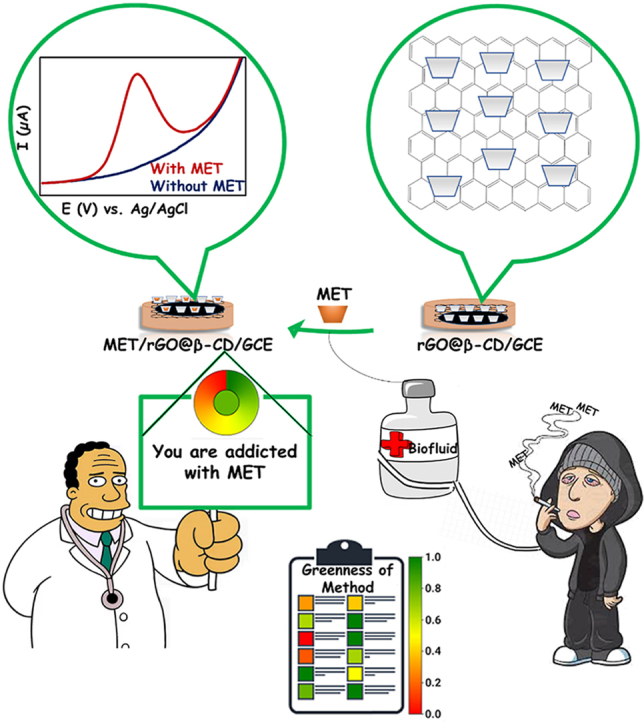

## 1 Introduction

Methadone (MET), scientifically known as (*RS*)-6-(dimethylamino)-4,4-diphenylheptan-3-one, is a synthetic μ-opioid receptor agonist classified as a group of powerful opioid maintenance therapy (OMT) [1]. Although the Food and Drug Administration (FDA) has approved this drug as pharmacotherapy for detoxification treatment of opioid dependence and for pain management, MET is often abused globally as a narcotic. The abuse of MET poses several serious risks, including of respiratory depression, divergence, delayed ventricular repolarization, dangerous interaction with other drugs. The most severe consequences can be fatal [2]. The estimated MET lethal dose varies depending on the individual's age and opioid tolerance. For children, it is approximately 32 μM; for opioid-naive adults, the range is between 96.94 μM and 161.57 μM; and for opioid-tolerant adults, it can exceed 387.78 μM–646.3 μM [3]. Since MET is involved in one-third of deaths due to opiate painkiller-related overdose, its close monitoring and level control in human biofluids is medically and judicially essential to diagnose poisoning or assist in the coroner's investigation of MET-induced death [4].

Various analytical methods based on chromatographic [[5], [6], [7], [8], [9], [10]] and spectrometric [11,12] instruments have been reported to identify MET. Despite the relative sensitivity and accuracy of some of them, limitations like sophisticated sample preparation, interferences of the response with biomolecules in the real sample, costly instrumentation and time-consuming analysis restrict their applicability. Some electrochemical sensors based on pencil graphite electrode (PGE) modified with multi-walled carbon nanotubes (MWCNTs) [13], carbon paste electrode (CPE) modified with gold nanofilm (AuNFs) [14] and poly-L-arginine/MWCNTs [15], glassy carbon electrodes (GCEs) modified with carbon quantum dots (CQDs)/MWCNTs [16] and mesoporous carbon/Ag nanoparticles (AgNPs)/graphene (Gr) [17] have issued several mentioned problems with various advantages such as faster and safer analysis. Nevertheless, these sensors are not user-friendly or durable enough for routine MET analysis in biofluids. Additionally, there is a main challenge in constructing an efficient and practical electrochemical sensor for biological matrices. Additionally, the development of an environmental-friendly sensor for a highly selective MET assay in saliva samples, with more boons like cost-effectiveness and commercialization capability is strongly demanded by clinical and legal organizations. Utilizing a durable green platform ensures the sensor's effectiveness in creating a stable and selective signal according to the principles of the green analytical chemistry (GAC) [[18], [19], [20]].

Cyclodextrins (CDs), from the family of cyclic oligosaccharides, are natural nanocapsules made up of glucose (Glu) derived from starch. Various types of these environmentally friendly nanoscale carriers (α-CD, β-CD, and γ-CD) can trap guest molecules inside their cavity via the inclusion of host-guest complexes by creating a hydrophobic zone via the central cavity of the cone lined with the skeletal carbons and ethereal oxygen of the glucose residues [21,22]. Since these microenvironments containing different sizes adapt to the shape and size of the guest, they are very selective for encapsulation [23]. β-CD is more widely considered for fabricating a selective sensor due to lower prices, better adaptation to the human body condition, and easier reactions than others [20,24]. The combination of β-CD with Gr derivatives with great interest the researchers in recent years [[25], [26], [27], [28], [29], [30], [31]], for the synthesis of the disulfide nanorods (SnS_2_)/Gr/β-CDs [32], graphene oxide (GO)/β-CDs [33,34], reduced GO/MWCNT/polyoxometalate (rGO/β-CD/MWCNT/POM) [35] and rGO (β-CD/rGO) [36,37] has provided high recognition capabilities for electrochemical sensor construction. It seems that integrating β-CD (as a green selective ligand) with GO [[38], [39], [40], [41], [42], [43], [44], [45]] (by a unique porous network structure) in the preparation of the beneficial rGO@β-CD nanocomposite is promising for high selective and sensitive recognition of MET in physiological conditions.

Our motivation in this study is the construction of the selective electrochemical sensor based on modifying the GCE by the rGO@β-CD nanocomposite for the determination of MET in human biofluids under the GAC concept, which has not been reported to date based on the best of our knowledge. The rGO not only increases the effective surface area and enhances electron transfer but also provides a porous network on the GCE surface to attach β-CD as a selective ligand toward MET in the proposed green nanocomposite. The sensor is able to catalyze MET at 0.8 V with a 7.76–fold current signal than the unmodified GCE. The feasibility of measuring MET in human biofluids with the developed sensor has been evaluated by analysis of human urine, plasma and saliva samples as real samples. The applied strategy has been satisfactorily appraised by the complimentary green analytical procedure index (ComplexGAPI) and the analytical greenness (AGREE) index as the common international criteria for assessing the greenness profile of the entire analytical methodology.

## 2 Experimental section

### 2.1 Materials

β-Cyclodextrin (β-CD), dopamine (DA), graphite, monosodium phosphate (NaH_2_PO_4_), sulfuric acid (H_2_SO_4_), hydrogen peroxide (H_2_O_2_), sodium nitrate (NaNO_3_), potassium chloride (KCl), ferro/ferricyanide ([Fe(CN)_6_]^3-/4-^), potassium permanganate (KnMO_4_), hydrazine (N_2_H_4_), sodium sulfate (Na_2_SO_4_), ammonia solution 25% (NH_3_), Glu, uric acid (UA) and ascorbic acid (AA) were purchased from Merck (Darmstadt, Germany) and Sigma Aldrich Companies (Burlington, Massachusetts, United States). Pure MET, acetaminophen (APAP), aspirin (ASA) and ibuprofen (IBP) were ordered from Temad Company (Tehran, Iran) and Raha Company (Isfahan, Iran). Methamphetamine (MAMP) was obtained from the Iranian Sanitation Ministry and the Center of Narcotics Prevention (Tehran, Iran). All materials were used without any purification and all solutions were prepared by double distillation (DI) water. 0.1 M of buffer phosphate (PB) with a pH value of 7 was used as a supporting electrolyte. 0.1 M KCl solution containing 5 mM [Fe(CN)_6_]^3-/4-^ was used as the redox probe for further electrochemical investigation.

### 2.2 Apparatus

All electrochemical measurements were performed by an Autolab Potentiostat/Galvanostat 302 N equipped with NOVA 2.4 software. A three-electrode system containing a platinum wire as the counter electrode (Metrohm company, Netherlands), Ag/AgCl (3M KCl) as the reference electrode (IV-EL company, China) and unmodified and modified GCE (2.5 mm of diameter) as the working electrode Azar electrode company, Iran) was used for recording the electrochemical signals. Cyclic voltammetry (CV), electrochemical impedance spectroscopy (EIS) and differential pulse voltammetry (DPV) techniques were applied to investigate the electrochemical behavior of the sensing interface.

The morphology, size, and elemental mapping of the synthesized rGO and rGO@β-CD were studied by a field effect scanning electron microscopy (FESEM) instrument (X-30 Philips, Netherlands) equipped with an energy-dispersive X-ray (EDX) component. The functional groups of the synthesized nanostructures were investigated by a Fourier-transform infrared (FTIR) spectroscopy via a PerkinElmer spectrophotometer instrument (United States).

The degree the greenness of the applied methodology was evaluated based on the metric systems including Complex GAPI and AGREE indexes by the freely available software on the following online links https://mostwiedzy.pl/complexgapi and https://mostwiedzy.pl/AGREE, respectively.

### 2.3 Synthesis of rGO@β-CD nanocomposite

GO was synthesized from graphite based on the well-known Hummers' method [42]. In a typical synthesis process, 2.0 g of graphite powder was dispersed in 140 mL of concentrated H_2_SO_4_, and 1.0 g of NaNO_3_ was added to them in an ice bath. Then, 6.0 g of KMnO_4_ was slowly added to the mixture under stirring (2 h) to fully oxidize graphite to GO and then, the resulting mixture was diluted with 140 mL of DI water. Next, 50 mL of H_2_O_2_ (5 % V/W) was added to the mixture until the color of the mixture changed to brilliant yellow. The suspension was filtered and the obtained GO was thoroughly washed with DI water. Afterward, GO was re-dispersed in DI water and exfoliated to generate nanosheets of the GO by ultrasonic bath for 3 h [46]. Then, 6 mL of the resulting GO suspension (2 mg mL^−1^) was mixed with 20 mL of β-CD (80 mg mL^−1^) and heated to 60 °C for 4 h. Then, 300 μL of NH_3_ (25 %) and 50 μL of N_2_H_4_ were added to the resulting mixture and heated at 60 °C for 4 h to reduce GO and produce the rGO. Finally, the resulting sediment, denoted as rGO@β-CD was centrifuged, and washed with DI water five times, and stored at room temperature for the next experiments. To check the control experiments, rGO was synthesized based on the same protocol in the presence of N_2_H_4_ and NH_3_ without β-CD.

### 2.4 The preparation of the electrochemical sensor for MET detection

**Scheme. 1:**
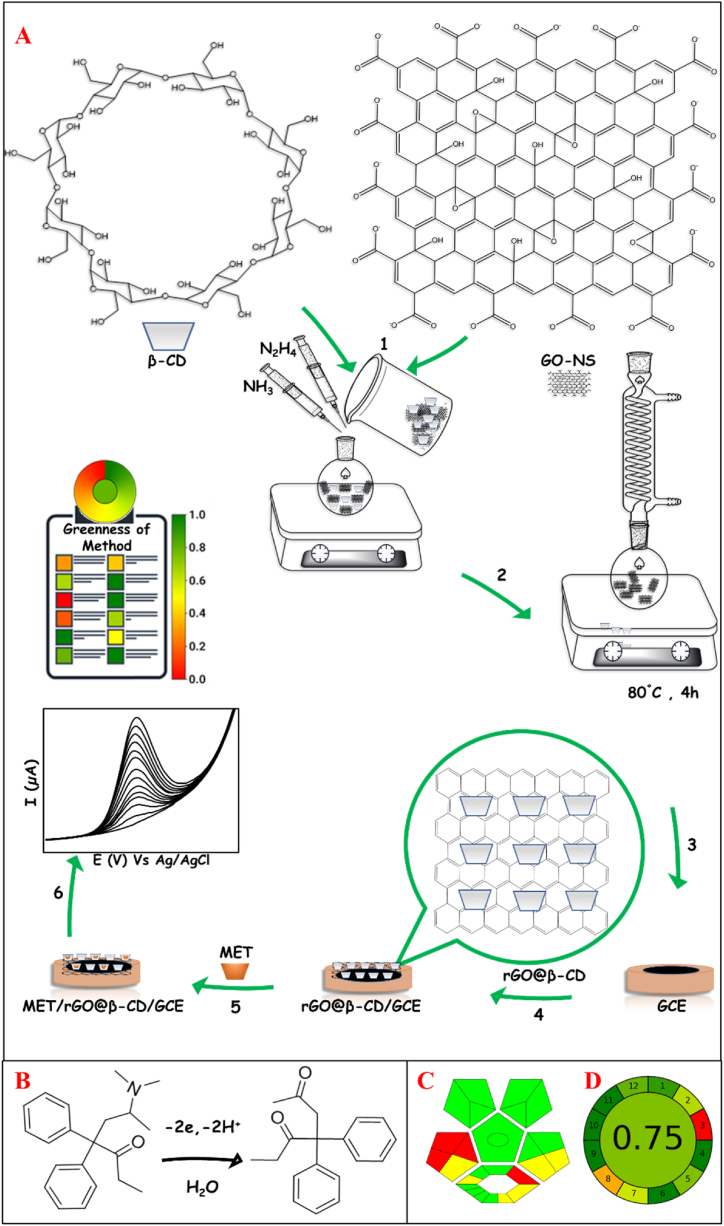
(A) The schematic representation of the fabrication process of the rGO@β-CD/GCE as the sensor and (B) the proposed mechanism for MET catalysis by the developed methodology. The resulting pictograms based on (C) ComplexGAPI and (D) AGREE indexes for evaluating of the greenness of the proposed method.

The GCE surface was polished with alumina and washed with DI water in an ultrasound bath to eliminate pollution and obtain a shiny electrode surface. Then, 7.5 μL of 7.5 mg mL^−1^ of the synthesized rGO@β-CD in DI water was drop-casted on the GCE surface and alloyed to dry at room temperature for 1 h. The GCE modified with rGO@β-CD nanocomposite, denoted as rGO@β-CD/GCE, was used as the working electrode for further experiments. A GCE modified with rGO, denoted as the rGO/GCE was prepared by the same protocol for control experiments. The developed sensor preparation process for the electrochemical detection of MET is schematically presented in Scheme. 1A.

## 3 Results and discussion

### 3.1 Characteristics of the synthesized rGO and rGO@β-CD

**Fig. 1:**
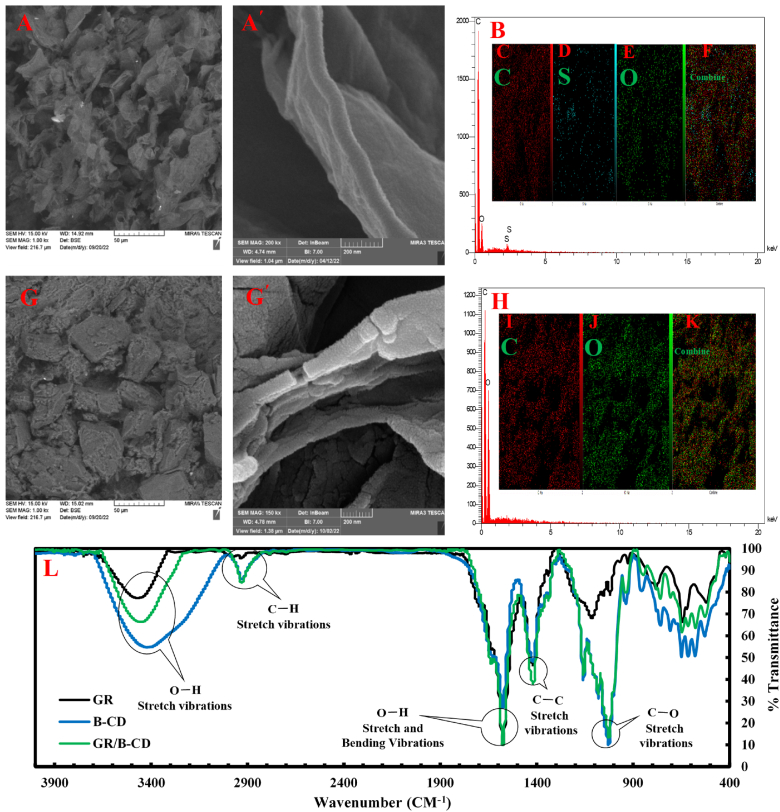
(A) and (A′) FESEM images with different magnifications and (B) EDX and map elemental analysis (C to F) of the synthesized rGO. (G) and (G′) FESEM images with different magnifications and (H) EDX and map elemental analysis (I to K) of the synthesized rGO/β-CD. (L) FTIR spectra of rGO, β-CD and rGO/β-CD.

The FESEM technique was used to characterize the morphology and surface features of the synthesized nanostructures (Fig. 1). FESEM images of rGO with different magnifications in Fig. 1A and A′ exhibit single flakes of the synthesized rGO with relatively large surfaces and the edge of sheets with the size of micrometers. This thin curtain-like morphology certifies a proper exfoliation of Gr during the oxidation process. The EDX and map elemental analysis in Fig. 1B–F indicates the presence of the oxygen (O), sulfur (S) and carbon (C) elements that prove the successful synthesis of GO according to the mentioned protocol. The investigation of FESEM images for the rGO/β-CD/GCE surface with different magnifications in Fig. 1G and G′ shows the oriented sheets stack randomly twist and wrap to form a fully connected porous framework. The β-CD inserted into the surface of the nanosheets relatively affects the initial thin curtain structure of rGO. Furthermore, the corresponding EDX (Fig. 1H) and map analysis (Fig. 1I–K) spectra containing peaks of O and C corroborate the insertion of β-CD within a porous carbon network.

The FTIR technique was applied to investigate the functional groups of the synthesized nanostructures (Fig. 1L). The rGO spectrum in Fig. 1L exhibits some absorbance peaks at 1580 cm^−1^ and 1190 cm^−1^, assigning to the stretching vibration of C=C and C–C/C–N bonds, respectively [47]. Several absorbance bonds at 2928 cm^−1^, 1716 cm^−1^ and 1440 cm^−1^ may be related to stretching vibration of the aromatic C–H, C=O and C–OH, respectively [48]. The β-CD spectrum indicates absorption peaks at 3400 cm^−1^ and 2928 cm^−1^ that may correspond to some stretching vibration of O–H and CH_2_ (methylene group), respectively. The sharp bonds at 1155 cm^−1^ may be related to antisymmetric stretching of the glycosidic C–O–C and O–H bending vibrations. Some absorption bands at 1030 cm^−1^ and 1076 cm^−1^ may be assigned to the coupled C–O/C–C stretching and O–H bending vibration of the saccharide structure of β-CD, respectively [49]. In rGO@β-CD spectrum, it can be observed that the absorbance bond at the region of 1155 cm^−1^ to 1030 cm^−1^ combines the featured peaks of β-CD and C–C/C–N stretching vibration bond of nanosheets, giving rise to the changes in intensity peak without the apparent peak shifts. These obvious changes in the intensity and location of some bonds in the rGO@β-CD spectrum compared to the three individual spectra confirm that β-CD has been successfully inserted into the nanosheet surface in the nanocomposite.

### 3.2 Electrochemical behavior of MET on the interface surface

**Fig. 2:**
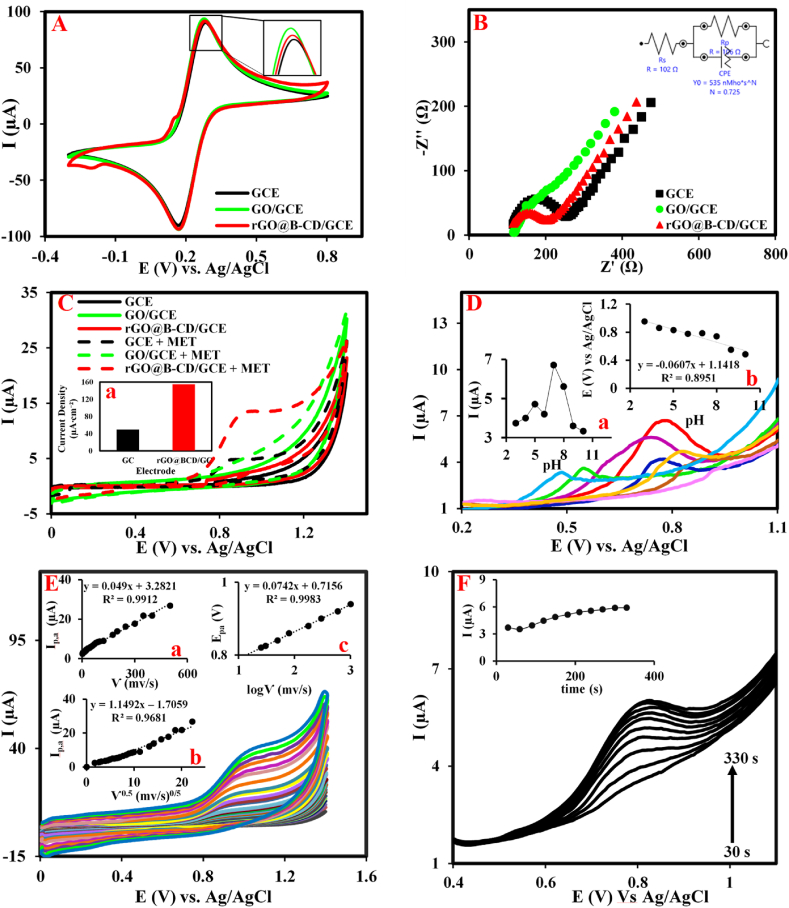
(A) CVs and (B) Nyquist curves along with the corresponding equivalent circuit for the unmodified GCE, rGO/GCE and rGO@β-CD/GCE in 5 mM of K_3_[Fe(CN)_6_]/K_4_[Fe(CN)_6_] and 0.1 M KCl as the redox probe. (C) CVs for the unmodified GCE, rGO/GCE and rGO@β-CD/GCE in the absence and presence of 250 μM MET in 0.1 M PB (pH = 7) at a scan rate of 100 mV s^−1^ (inset: histogram of the current density for the GCE and rGO@β-CD/GCE surfaces). (D) The recorded DPVs of rGO@β-CD/GCE in 0.1 M of PB at a pH range from 3 to 10 in the presence of 30 μM of MET, (E) CVs of rGO@β-CD/GCE in 0.1 M of PB (pH = 7.0) containing 250 μM of MET at a scan rate range of 3 mV s^−1^ to 500 mV s^−1^ (insets: the corresponding plots for (a) I_p,a_ vs. *ν*, (b) I_p,a_ vs. *ν*^0.5^ and (c) E_p,a_ vs. log *ν*). (F) DPVs of rGO@β-CD/GCE in 0.1 M of PB (pH = 7.0) containing 10 μM of MET in the accumulation time range of 30 s–300 s (inset: the corresponding plots for (a) I_p,a_ vs. time).

As mentioned before, the rGO@β-CD nanocomposite for modification of the GCE surface plays a pivotal role in the sensor construction. To examine the electrochemical behavior of the modified GCE surface the CV technique was used and corresponding cyclic voltammograms of unmodified GCE, rGO@β-CD/GCE and rGO/GCE were recorded in 0.1 M KCl solution containing 5 mM [Fe(CN)_6_]^3-^/^4-^ as the redox probe (Fig. 2). As shown in Fig. 2A, the unmodified GCE presents a well-defined peak related to the redox couple while GO attachment on the surface in the presence of the reluctant agents (rGO/GCE) increases the current signal without any distinguished replacement of peak-to-peak potential separation (ΔE_p_). The rGO@β-CD formation on the GCE surface slightly decreases the current signal, which is attributed to the low spatial hindrance created by β-CD on the surface. Noteworthy, one of the most important differences between GO and rGO is the electrical conductivity such that rGO presents better electrical conductivity than GO. Although it is expected that rGO provides a better electron transfer on the rGO@β-CD/GCE surface the presence of massive β-CD with more spatial hindrance decreases the electron transfer ability [37]. To appraise the modified electrode surface the EIS technique was applied and Nyquist curves were recorded in 0.1 M KCl solution containing 5 mM [Fe(CN)_6_]^3-^/^4-^ as the redox probe in a frequency range of 10 to 0.1 kHz at 220 mV. In this technique, the change in diameter of the obtained semicircle that is proportional to the charge transfer resistance (R_ct_) value implies the kinetic behavior of the anion probe on the modified electrode interface to confirm the attachment of each layer on the GCE surface during modiﬁcation steps. Nyquist plots in Fig. 2B present a capacitive semicircle with an R_ct_ value of 149 Ω for the unmodified GCE, while the R_ct_ value is decreased to 68 Ω for the rGO/GCE surface. This behavior is attributed to the high electron transfer feature of the porous rGO layer. The increasing of the R_ct_ value to 161 Ω in the rGO@β-CD/GCE surface reveals the attachment of the rGO@β-CD layer by further spatial hindrance on the GCE surface that prohibits the diffusion of the redox probe to the electrode surface. It seems the presence of the β-CD with more spatial hindrance overcomes the electron transfer ability of the conductive rGO in the rGO@β-CD/GCE surface [37] and decreases the electron transfer of the redox couple on the interface surface. Both CV and EIS techniques corroborate the correct attachment of the layers in the sensor fabrication process.

CV technique was used to investigate the ability of the modified GCE for the electrochemical oxidation of MET (Fig. 2C). Fig. 2C shows voltammograms of the unmodified GCE, rGO/GCE, and rGO@β-CD/GCE in the absence and presence of MET (250 μM) in 0.1M PB (pH = 7) at a scan rate of 100 mV s^−1^. Comparing the unmodified GCE in the absence and presence of MET indicates MET is slightly catalyzed on the unmodified GCE at 0.65 V with a current intensity of 4 μA. Although rGO/GCE as the control sample does not exhibit any current signal in the presence of MET and only enhances the capacitive current, the rGO@β-CD/GCE remarkably catalyzes MET at 0.65 V with current intensity (13 μA), which is approximately 3.2-folds more than unmodified GCE. This behavior reveals that the rGO@β-CD/GCE as the sensor has an excellent capability in MET catalysis which is attributed to the synergetic effect of the features related to the nanosheets and β-CD used in the proposed nanocomposite onto the GCE surface.

To study the effect of the rGO@β-CD on the increase of the electron transfer rate of the modified GCE, the surface area (*A*, cm^2^) value was calculated by the Randles Sevcik equation (Eq.) for the reversible process of the redox probe on the surface at various scan rates according to Fig. 1S [20]. Considering the obtained slopes for the unmodified GCE and rGO@β-CD/GCE in the inset of Fig. 1S–A and B, the values of *A* were calculated to be 0.08 cm^2^ and 0.087 cm^2^, respectively. These values indicate that the β-CD molecules with a big size overcome the natural ability of the rGO in the surface area enhancement and the rGO@β-CD/GCE makes a bit more surface area than the unmodified GCE surface.

### 3.3 pH effect

The acidity effect of the electrolyte solution on the oxidation current of MET was investigated at a pH range from 3 to 10 in the 0.1 M of PB containing 30 μM of MET by DPV technique (Fig. 2D). As depicted in Fig. 2D, the oxidation current intensity of MET changes with the pH value increasing and indicates a maximum current at the pH value of 7 (inset a). This behavior reveals that the catalysis of MET by the proposed sensor needs a neutral condition and an alkaline or acidic condition decreases the sensor's capability for MET detection. Thus, the pH value of 7 as the optimized pH was selected in the subsequent experiment. One of the advantages of this study is the capability of MET measurement at pH = 7 as the physiologic pH, which is very helpful in MET measurement in human biofluids. Additionally, the results reflect that the peak potential values are displaced to the less positive potentials with the increasing pH value as shown in inset b of Fig. 2D. Accordingly, the linear relationship between E_p,a_ and pH values for MET oxidation is estimated by the E_p,a_ = −0.0607 pH + 1.1418. Using Eq. (1) [50], slops of 60 mV pH^−1^ are estimated for the oxidation reaction of MET which indicates the contribution of equal numbers of the involved protons and electrons in the oxidation reaction of MET [50]. Consequently, the proposed mechanism in Scheme. 1B is predicted for the electrochemical oxidation of MET according to the literature [51,52].

(Eq. 1) $$E^{ᵒ´}=E^{ᵒ}-\frac{\left(0.0592m\right)}{\text{nF}}\text{pH}$$

### 3.4 Effect of potential scan rate

To evaluate how the mass transfer process takes place on the developed sensor surface the electrochemical behavior of the rGO@β-CD/GCE was investigated by recording the corresponding CVs in the presence of 250 μM of MET at different scan rates. As illustrated in Fig. 2E, the anodic peak current (I_p,a_) of the oxidation process of MET on the sensing interface is remarkably increased by scan rate increasing from 3 mV s^−1^ to 500 mV s^−1^, while no reduction peak current is observed in the reverse scan in all investigated sweep rates. According to the inset a in Fig. 2E–a good linear relationship between I_p,a_ and scan rate (*ν*) renders a non-diffusion current in the MET catalysis. This electrochemical behavior may be rooted in the fact that the oxidation reaction mechanism of the MET is based on the adsorption process on the sensor surface [53]. According to Laviron's theory [54], the relation between I_p,a_ and *ν* can be described by Eq. (2), rendering an adsorbed surface-controlled process for the oxidation of MET by the proposed sensor:

(Eq. 2) $$i_{p}=\frac{n^{2}F^{2}ᴦA\nu }{4RT}=\frac{nFQ\nu }{4RT}$$

(Eq. 3) $$E_{pa}=\frac{b}{2}\log\;\nu +constant$$

(Eq. 4) $$b=\frac{2.303\;RT}{\left(1-\alpha \right)nF}$$

where, A is the active surface area of the electrode, n is the number of the transferred electrons, F is the Faraday constant and all other symbols have the normal meaning. The amount of b and α was calculated to be 0.1484 and 0.8, according to Eq. (3) and Eq. (4) and the obtained slope (0.1484) from the plot of E_p,a_ versus log *ν* (inset c in Fig. 2E), presenting good oxidation of MET on the electrode surface based on the adsorbed surface-controlled processes.

### 3.5 Accumulation time effect

The possibility of the MET adsorption on rGO@β-CD/GCE surface was appraised by recording DPV curves in 0.1 M PB (pH = 7) containing 10 μM of MET under 30 s–300 s as accumulation time (Fig. 2F). The I_p,a_ value was remarkably increased with the pre-concentration time increasing from 30 s to 330 s to reach a topmost value at 300 s while the peak current stayed unchanged with more increases in the accumulation time. So, 300 s was selected as the optimum accumulation time in all experiments.

### 3.6 Voltammetric determination of MET

**Fig. 3:**
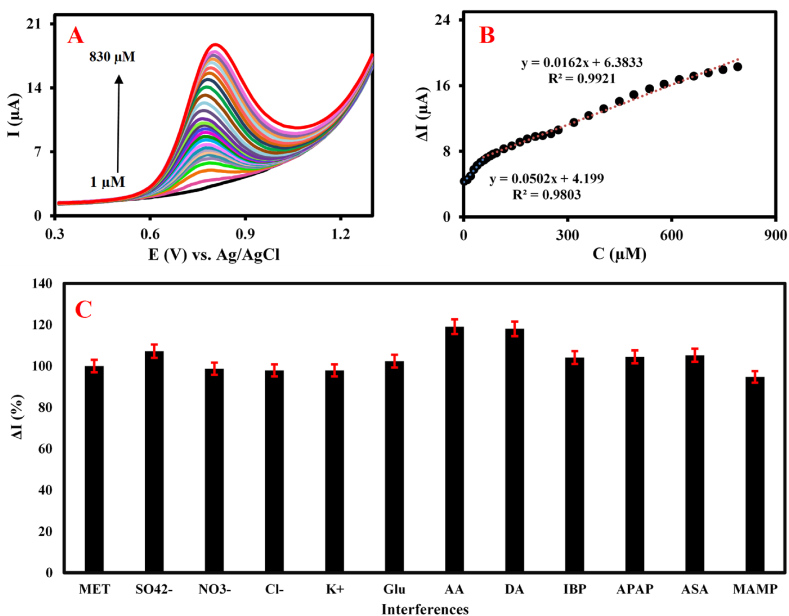
(A) The recorded DPVs of the developed sensor in 0.1 M PB (pH = 7) containing different concentrations of MET from 1 μM to 830 μM and (B) two corresponding linear relationships for the ΔI_p,a_ vs. different concentrations of MET. (C) The bar charts extracted from DPVs of the developed sensor toward some common interfering species and ions including 300 μM of Glu, IBP, MAMP, ASA, APAP, SO_4_^2−^, NO_3_^−^, Cl^−^ and K^+^ and 30 μM of DA and AA in the presence of 30 μM of MET in 0.1 M PB (pH = 7).

Different concentrations of MET in 0.1 M PB (pH = 7) were tested to evaluate the efficiency of the rGO@β-CD/GCE as the electrochemical sensor (Fig. 3A). As plotted in Fig. 3B, the corresponding DPVs are regularly increased by adding MET concentrations from 1 μM to 830 μM under two regression relationships including ΔI (μA) = 0.0502 C_MET_ + 4.199 with R^2^ = 0.9803 (for 1 μM–57.5 μM) and ΔI (μA) = 0.0162 C_MET_ + 6.3833 with R^2^ = 0.9921 (for 57.5 μM–830 μM). The limit of detection (LOD) value and analytical sensitivity were calculated to be 333.33 nM and 0.0502 μA μM^−1^, respectively. A comparison between the results of this sensor with some reported MET sensors in Table 1 exhibits that the developed strategy in this study supports a broad linear dynamic range (LDR) value, which includes all reported lethal dose ranges of MET for abusers of different ages. These satisfactory results can be attributed to the synergetic effect of rGO and β-CD properties in the proposed nanocomposite in the GCE surface modifying process.

**Table 1:** Comparison of the results of the proposed sensor and other sensors for MET analysis based on various sensing interfaces and different matrices.

Method	Sensing Interface	LDR (μM)	LOD (nM)	Analyzed medium	Ref.
**Non-electrochemical Methods**					
GC-MS	–	–	–	Urine/Plasma	[5]
GC-FID	MIP	0.012 to 129.26	0.008/0.007	Plasma/Saliva	[9]
DLLME–HPLC–UV	–	0.032 to 16.15	0.015/0.023/0.081/0.080	Urine/Plasma/Saliva/Sweat	[7]
GC	CNT-EME	–	4.84 × 10^−9^	Urine/Plasma	[8]
HPLC–MS/MS	–	1.61 × 10^-4^ to 3.23	3.23 × 10^-4^	Plasma/Saliva	[6]
AAS	–	0.016 to 0.161	–	Urine/Tablet	[10]
CE–ECL	(Ru(bpy)_3_^2+^)	5 to 200	500	–	[11]
ECL	Ru(bpy)_3_^2+^/Yb_2_O_3_ NPs/Nf/CPE	0.005 to 0.7	0.21	Urine	[12]
**Electrochemical Methods**					
DPV	MWCNT-PGE	0.1 to 15	0.87	Urine/Plasma	[13]
SWV	GNPs/MWCNT-CPE	0.1 to 500	5	Urine/Saliva	[14]
DPV	CQDs-MWCNTs	0.1 to 225	30	Urine/Plasma	[16]
SWV	CMK-5	0.1 to 4	29	Urine	[55]
DPV	(Gr/AgNPs)_2_-GCE	1 to 200	120	Plasma	[17]
DPV	TGA@CdSe/GO	0.1 to 20 & 20 to 323	30	Plasma	[56]
Amperometry	P-L-Arg/CNTs-CPE	0 to 120	22	Urine	[15]
DPV	Ox-p(Thp)/AuNPs/CC	0.049 to 9.9 & 9.9 to 29	14	Urine/Plasma	[53]
DPV	rGO@β-CD/GCE	1 to 830	333.3	Urine/Plasma/Saliva	This study

GC-MS (Gas chromatography–mass spectrometry), GC-FID (Gas chromatography-flam ionization detector), MIP (Molecularly imprinted polymer), DLLME–HPLC–UV (dispersive liquid–liquid microextraction-high performance liquid chromatography with ultra violet), GC (Gas chromatography), HPLC–MS/MS (High performance liquid chromatography-mass spectrometry/mass spectrometry), AAS (Atomic absorption spectrometry), CE–ECL (Capillary electrophoresis with electrochemiluminescence), Ru(bpy)_3_^2+^ tris(2,2**′**-bipyridyl) ruthenium(II), ECL (Electrochemiluminescence), Ru(bpy)_3_^2+^/Yb_2_O_3_NPs/Nf (tris(2,2**′**-bipyridyl) ruthenium(II)/ytterbium oxide nanoparticles/Nafion/carbon paste electrode), DPV **(**Differential pulse voltammetry), MWCNT/PGE (multiwalled carbon nanotube modified pencil graphite electrode), SWV (square wave voltammetry), GNPs/MWCNT-CPE (gold nanoparticle multiwalled carbon nanotube modified carbon paste electrode), CMK-5 (a group of ordered mesoporous carbon with high porosity and a large surface area), Gr/AgNPs-GCE (glassy carbon electrode modified with two layers of graphene/Ag nanoparticles), TGA@CdSe/GO (Thioglycolic Acid Decorated CdSe Doped GO Multilayers), P-L-Arg/CNTs-CPE (poly-larginine/carbon nanotubes composite modified carbon paste electrode), Ox-p(Thp)/AuNPs/CC (gold nanoparticles/polythiophene modified carbon cloth platform).

### 3.7 Interference, repeatability, reproducibility and stability investigation

Some electroactive compounds and ions present in most human biofluids as the common interfering species, which are susceptible to causing disturbances in the real measurements, were tested by the developed sensor in 0.1 M PB (pH = 7) under optimal conditions. The bar chart extracted from the DPVs related to the sensor in Fig. 3C reveals that the species including 300 μM of Glu, IBP, MAMP, ASA, APAP, SO_4_^2−^, NO_3_^−^, Cl^−^ and K^+^ (with a 10-fold concentration than MET) and 30 μM of DA and AA (with the same concentration than MET) did not interfere with the measurement of 30 μM of MET. These findings present satisfactory anti-interference potentiality of the sensor in MET sensing. The repeatability of the sensor was checked by recording DPVs in the presence of 25 μM of MET for triplicate. The relative standard deviation (RSD) value of 3.71 % confirms the good repeatability of the applied strategy (data not shown). The reproducibility of the proposed methodology was confirmed by testing seven fabricated rGO@β-CD/GCEs and recording the corresponding DPVs in 0.1 M PB (pH = 7) containing 50 μM of MET based on the satisfactory RSD value of 2.40 % (data not shown). Moreover, the storage stability parameter was evaluated by storing the sensor at 4 °C and determining 100 μM of MET by DPV technique for one month. The weekly monitoring of the oxidation peak current of MET every week indicated the sensor retained 89 % of the initial current, revealing acceptable storage stability in the analysis of MET (data not shown). These satisfactory results in MET sensing by the proposed sensor may be rooted in some key factors; namely (1) utilizing rGO with fantastic properties in active surface area enhancement and tunneling electron transfer improvement and (2) using environmentally friendly β-CD by creating a central cavity to fitly trap MET based on its shape and size and boost selective detection of MET.

### 3.8 Real sample analysis

To evaluate the feasibility of MET analysis in real samples by the developed sensor some human biofluids were tested. The urine, plasma and saliva samples from some fasting healthy men were randomly collected from a local clinic at Marvdasht City according to ethical principles. The ethics ID IR was assigned with *IR.YUMS.BLC.1403.011* approved by Biosafety and Laboratory Standards Committee-Yasouj University of Medical Sciences (https://ethics.research.ac.ir/IR.YUMS.BLC.1403.011). Each sample was separately centrifuged (10 min, 3000 rpm) and filtered. The samples were individually diluted with 0.1 M of PB (pH = 7) at a ratio of 1:1 and used as the electrolyte for the detection of different concentrations of MET as follows:

#### 3.8.1 MET determination in the urine sample

**Fig. 4:**
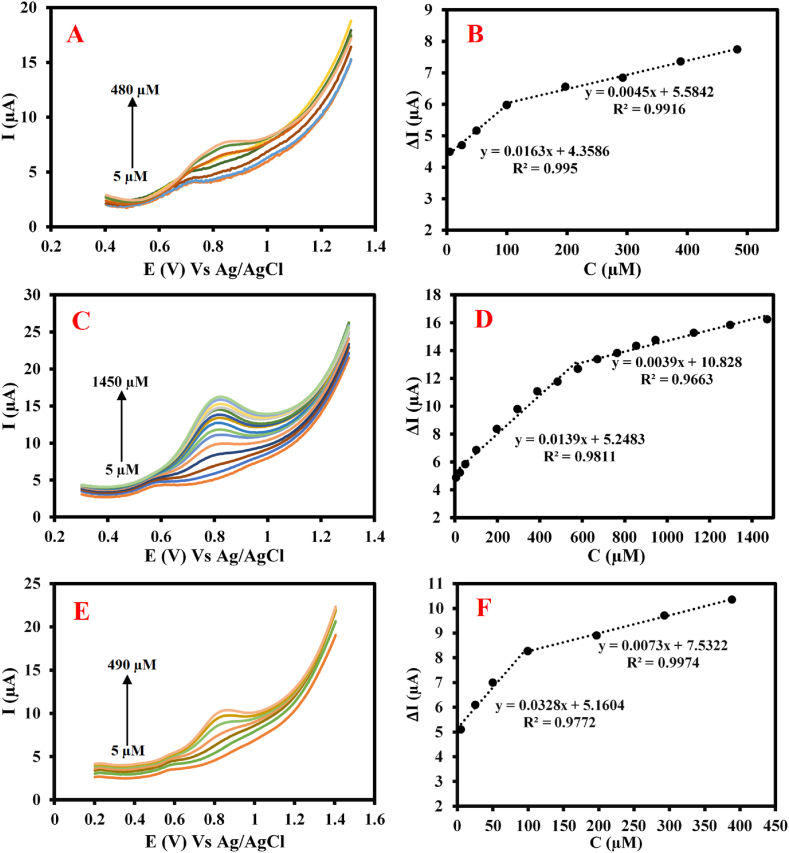
The recorded DPVs of the sensor for the prepared (A) urine sample containing different concentrations of MET (5 μM–480 μM), (C) serum sample containing different concentrations of MET (5 μM–1.470 mM), (E) saliva sample containing different concentrations of MET (5 μM–490 μM) along with (B, D and F) two corresponding calibration curves (all samples were diluted by 0.1 M PB (pH = 7) at a ratio of 1:1).

A series of different concentrations of MET from 5 μM to 480 μM was separately injected into the prepared urine sample as the electrolyte and the corresponding DPVs were recorded (Fig. 4A). According to Fig. 4B, the current changes are linearly increased by increasing concentrations of MET from 5 μM to 480 μM under two regression relationships including ΔI (μA) = 0.0163 C_MET_ + 4.358 with R^2^ = 0.995 (for 5 μM–100 μM) and ΔI (μA) = 0.0045 C_MET_ + 0.5842 with R^2^ = 0.9916 (for 100 μM–480 μM). The LOD and analytical sensitivity values for MET sensing in the urine sample were calculated to be 166.7 nM and 0.0163 μA μM^−1^, respectively.

#### 3.8.2 MET determination in the serum sample

A series of different concentrations of MET from 5 μM to 1.470 mM were separately injected into the prepared serum sample as the electrolyte and the corresponding DPVs were recorded (Fig. 4C). As plotted in Fig. 4D, the current changes are regularly increased by increasing concentrations of MET from 5 μM to 1.470 mM under two regression relationships including ΔI (μA) = 0.0139 C_MET_ + 5.2483 with R^2^ = 0.9811 (for 5 μM–575 μM) and ΔI (μA) = 0.0039 C_MET_ + 10.828 with R^2^ = 0.9663 (for 575 μM to 1.470 mM). The LOD and analytical sensitivity values for MET detection in the prepared serum sample were calculated to be 166.7 nM and 0.0139 μA μM^−1^, respectively.

#### 3.8.3 MET determination in the saliva sample

A series of different concentrations of MET from 5 μM to 490 μM was separately injected into the prepared saliva sample as the electrolyte and the corresponding DPVs were recorded (Fig. 4E). As shown in Fig. 4F, the signal changes are increased by adding MET concentrations from 5 μM to 100 μM under two regression relationships including ΔI (μA) = 0.0328 C_MET_ + 5.1604 with R^2^ = 0.9977 (for 5 μM–100 μM) and ΔI (μA) = 0.0039 C_MET_ + 10.828 with R^2^ = 0.9663 (for 100 μM–490 μM). LOD and analytical sensitivity values for the measuring MET in the saliva sample were calculated to be 166.7 nM and 0.0328 μA μM^−1^, respectively.

These satisfactory results for MET sensing in the various biofluids by the proposed sensor exhibit the acceptable applicability of the sensor for urine, serum and saliva analysis. The successful tracing of MET in saliva samples promises the applicability of the efficient sensor for non-invasive and readily assay of MET.

### 3.9 Greenness profile of the proposed method

The GAC concept focuses on providing analytical methodologies with safer and eco-friendly indexes for the environment and humans. The required energy amount, purification and toxicity of chemicals and reagents, produced waste, miniaturization and automation ability, yield and the number of procedural steps are key items considered for evaluating the greenness of an analytical procedure [57]. One of the common international metric systems for appraising the greenness profile of one method is the ComplexGAPI index which provides an output pictogram based on the different greenness degrees from low to medium and high risks based on the green, yellow and red color, respectively. These colorimetric degrees are defined based on the entire experimental process from sample preparation to the latest analysis steps within a hexagonal area [58,59]. Another international scoring system for evaluating environmental friendliness according to the twelve principles of the GAC is the AGREE index. By converting each principle into a numerical value in this metric system a circular pictogram resulted in 12 sub-parts that present risk indicators by a final score from 0 to 1 with various colors from green to red [60].

The evaluation of the degree of greenness of the applied strategy according to the input condition via ComplexGAPI and AGREE metrics confirms the greenness of the proposed methodology from an eco-friendly view, as evidenced in the resulting pictograms in Scheme. 1C and D, respectively. The reasons behind this matter are mainly attributed to the use of B-CD as an environmental-friendly reagent and other non-toxic and cost-effective chemicals, the minimal quantity of chemicals according to the atomic economy theory, using less energy, the miniaturization and commercialization ability of sensor for MET assay.

## 4 Conclusion

The selective and cost-efficient sensor was introduced based on GCE modified with rGO@β-CD for real-time electrochemical detection of MET. The assay principle mainly relied on the oxidation of MET on the proposed sensing interface under the physiological pH condition upon the analyte addition. Utilizing β-CD enhanced the selectivity parameter of the sensor by trapping MET based on its shape and size on the sensing interface toward other common interfering species. The sensor exhibited good LOD and LDR values for MET detection, which included a reported concentration level based on the opioid tolerance of consumers according to the literature. One of the advantages of this study is the feasibility of real-time and non-invasive detection of MET by the proposed sensor in various human biofluids and the calculation of the LDR and LOD values in the samples based on the GCA concept compared to other reported MET sensors. Additionally, the greenness performance of the proposed strategy was well evaluated according to the GCA principles based on the common international indexes because utilization of the green and eco-friendly nanocomposite, low amount of chemicals and energy as well as presenting a simple and fast strategy without sophisticated preparation samples. Since quick monitoring of MET is essential in human biofluids from the clinical and judicial view, the proposed strategy may be a convenient method for the real-time and non-invasive distinction of narcotics in suspicious people biofluids.

## CRediT authorship contribution statement

**Sayyed Esmaeil Moradi:** Writing – original draft, Software, Formal analysis, Data curation. **Ardeshir Shokrollahi:** Validation, Project administration. **Faezeh Shahdost-Fard:** Writing – review & editing, Supervision.

## Ethics ID

The ethics ID IR was assigned with *IR.YUMS.BLC.1403.011*. The link to view the approval online can be found on the website of the National System of Ethics in Biomedical Research at the following address at https://ethics.research.ac.ir/IR.YUMS.BLC.1403.011 approved by Biosafety and Laboratory Standards Committee - Yasouj University of Medical Sciences.

## Declaration of Competing Interest

The authors declare the following financial interests/personal relationships which may be considered as potential competing interests: Faezeh Shahdost-Fard reports financial support and equipment, drugs, or supplies were provided by Yasouj University. Ardeshir Shokrolahi reports a relationship with Yasouj University that includes: board membership. If there are other authors, they declare that they have no known competing financial interests or personal relationships that could have appeared to influence the work reported in this paper.
