# Supplementary material for: Applicability of a green nanocomposite consists of reduced graphene oxide and β-cyclodextrin for electrochemical tracing of methadone in human biofluids validated by international greenness indexes
Source: Heliyon. 2024 Nov 19;10(23):e40505. doi: 10.1016/j.heliyon.2024.e40505 (PMC11636103; doi:10.1016/j.heliyon.2024.e40505)
Supplement: Multimedia component 1 [file mmc1.docx]

**Supporting Information**

**Applicability of a green nanocomposite consists of reduced graphene oxide and β-cyclodextrin for electrochemical tracing of methadone in human biofluids validated by international greenness indexes**

Sayyed Esmaeil Moradi^1^, Ardeshir Shokrollahi^1,*^, Faezeh Shahdost-Fard^2,*^

*^1^ Chemistry Department, Yasouj University, Yasouj 75918-74831, Iran*

*^2^ Department of Chemistry Education, Farhangian University, P.O. Box 14665-889, Tehran, Iran*

^*^Corresponding authors: E-mail address and Numbers: [ashokrollahi@yu.ac.ir](mailto:ashokrollahi@yu.ac.ir), +989173232713 (A. Shokrollahi) and [F.Shahdost@gmail.com](mailto:F.Shahdost@gmail.com) and [Shahdost@cfu.ac.ir](mailto:Shahdost@cfu.ac.ir) , +989138285755 (F. Shahdost-Fard)

Fig. S1. (A) and (B) CVs of the GCE and rGO@β-CD/GCE in 5 mM of K_3_[Fe(CN)_6_]/K_4_[Fe(CN)_6_] and 0.1 M KCl as the redox probe (insets: I_p_ vs. ν^0.5^).
